# Comprehensive Study on the Mechanism of Sulfating Roasting of Zinc Plant Residue with Iron Sulfates

**DOI:** 10.3390/ma14175020

**Published:** 2021-09-02

**Authors:** Pavel Grudinsky, Denis Pankratov, Dmitry Kovalev, Darya Grigoreva, Valery Dyubanov

**Affiliations:** 1 Laboratory of Physical Chemistry and Technology of Iron Ore Processing, A.A. Baikov Institute of Metallurgy and Materials Science, Russian Academy of Science, 49 Leninsky Prosp., 119334 Moscow, Russia; dashagrgrv121@gmail.com (D.G.); vdyubanov@imet.ac.ru (V.D.); 2 Department of Radiochemistry, Faculty of Chemistry, Lomonosov Moscow State University, 1-3 Leninskiye Gory, 119991 Moscow, Russia; pankratov@radio.chem.msu.ru; 3 Department of X-ray Investigation, Merzhanov Institute of Structural Macrokinetics and Materials Science (ISMAN), 8 Academician Osipyan Street, 142432 Chernogolovka, Moscow Region, Russia; kovalev@ism.ac.ru

**Keywords:** zinc plant residue, zinc ferrite residue, zinc ferrite, copper ferrite, iron sulfate, sulfating roasting, Mössbauer spectroscopy, time-resolved X-ray diffraction, chemical phase analysis

## Abstract

Zinc plant residue (ZPR) is a secondary material generated during hydrometallurgical zinc production that contains considerable contents of valuable elements such as Zn, Cu, Fe, Pb, Cd, Ag, In, Ga, Tl. Zinc, copper and accompanying elements in ZPR are in different minerals, mainly in the ferrites. A promising approach for recycling ZPR is the sulfating roasting using iron sulfates followed by water leaching. In this study, the composition of ZPR and the obtained products were thoroughly investigated by various methods including X-ray diffraction analysis (XRD), chemical phase analysis and Mössbauer spectroscopy. The effect of temperature, amount of iron sulfates and roasting time on the conversion of valuable metals into a water-soluble form was thermodynamically and experimentally studied both using pure ferrites and ZPR. Based on the results of time-resolved XRD analysis and synchronous thermal analysis (STA), a mechanism of the sulfation roasting was elucidated. The rate-controlling step of zinc and copper sulfation process during the ZPR roasting was estimated. The sulfating roasting at 600 °C during 180 min with the optimal Fe_2_(SO_4_)_3_∙9H_2_O addition followed by water leaching enables to recover 99% Zn and 80.3% Cu, while Fe, Pb, Ag, In, Ga retained almost fully in the residue.

## 1. Introduction

Zinc complex sulfide ores are the main raw materials for zinc production that contain associated components such as lead, copper, cadmium, silver, gold, bismuth, indium, gallium and thallium [1]. Therefore, zinc production generates intermediate products and wastes with significant contents of non-ferrous metals that are considerably higher than the contents in the ore. Due to a depletion of deposits of non-ferrous metals ores and a decrease of ore grade [2], as well as the need to solve environmental problems associated with the storage of highly toxic metallurgical wastes in dumps [3,4], it is necessary to recycle the generated materials.

Currently, more than 90% of zinc is produced by the hydrometallurgical route [5] that includes oxidative roasting in fluidized-bed furnaces, sulfuric acid leaching and electrolysis. The process of the roasting of zinc sulfide concentrates is a substantially cost-effective technology due to its autogenicity, a satisfactory removal of harmful impurities for the subsequent electrolysis and the associated production of sulfuric acid [6] (pp. 38–54). However, during the roasting, besides highly soluble zinc oxide, a certain amount of zinc and other valuable non-ferrous metals reacts with iron to obtain ferrites of a low solubility. It necessitates the application of additional stages after the leaching of zinc oxide to extract the remaining ferritic zinc. Currently, the most widely used method is the leaching of the residue by strong hot sulfuric acid with following precipitation of iron in the form of jarosite [7]. Other industrial methods are the same leaching with the precipitation of iron as goethite or hematite [8], as well as the pyrometallurgical processing of the ferrite residue by the Waelz process [9,10].

Some authors have studied other leaching methods for the processing of zinc ferrite residue by sulfuric acid [11,12,13], di-2-ethylhexyl phosphoric acid [14], sodium hydroxide [15,16], chloride solutions [17,18,19], and even by ionic liquids [20] and deep-eutectic solvents [21]. The methods using pyrometallurgical processing of such materials by reduction roasting [22,23,24,25], sulfiding roasting [26,27], roasting with the addition of sodium compounds [28,29,30,31], chloride volatilization process [32] were also reported. However, these methods are still hardly applicable in the industrial practice.

Separately, methods should be noted to obtain water-soluble zinc sulfate by the roasting with elemental sulfur in air [33], sulfuric acid [34,35,36], ammonium sulfate [37,38], as well as using blowing of mixture of sulfur dioxide and air [39,40]. Sulfation method has several advantages compared with other methods. The main advantages are relatively low roasting temperature range, lack of fuel consumption due to exothermic sulfating reactions [41], the application of water leaching rather than acid leaching after the roasting to extract zinc and other valuable components. Some authors have studied iron sulfates as sulfating agents for roasting of zinc ferrite residues [42,43], copper slag [44,45], copper tailings [46,47] and electric arc furnace dust from steelmaking [48].

This paper focuses on the investigation of the mechanism of zinc and copper sulfation, as well as a behavior of iron, cadmium, lead, silver, indium, gallium and thallium during the roasting of zinc plant residue (ZPR) with iron sulfates. The study presents the results of multifactorial research of the effect of temperature, roasting time and amount of iron sulfates on the conversion of non-ferrous metals into water-soluble form both using pure zinc and copper ferrites, as well as ZPR.

## 2. Materials and Methods

### 2.1. Raw Materials

Chemically pure reagents FeSO_4_∙7H_2_O and Fe_2_(SO_4_)_3_∙9H_2_O were used in the experiments. Ferrites were prepared from compounds of analytical grade. A V-shell blender treatment with a duration time of 24 h was used to prepare powder mixtures of raw materials.

Pure zinc ferrite ZnFe_2_O_4_ was prepared by the following method. Pure Fe_2_O_3_ and ZnO were mixed in a 1.0:1.1 molar ratio with a 10% excess of zinc oxide and briquetted by hand hydraulic press. The briquettes from each 1 g of the mixtures were prepared using a mold of 17 mm in diameter; the used pressure was 200 MPa. The briquettes were calcined in a muffle furnace at 1000 °C for 6 h in air. The calcined sample was ground and sieved up to the fraction of 0.2 mm. Then the residual free zinc oxide was leached from the calcine by ammonium chloride solution containing 22 g NH_4_Cl in 200 g of 14% NH_4_OH at 50–60 °C for 2 h [49] (p. 111). The leached residue was calcined again in the muffle furnace at 1000 °C for 2 h. The obtained ferrite powder was grinded, sieved, and then it was tested for impurities by X-ray diffraction (XRD) analysis.

Pure copper and zinc-copper ferrites were prepared according to the method [50]. Pure reagents Fe_2_O_3_, CuO and ZnO were weighed in exact molar ratios to obtain the corresponding ferrites CuFe_2_O_4_ and Zn_0.5_Cu_0.5_Fe_2_O_4_. The mineralizer (1% KCl) was also added to oxide components. After mixing and briquetting, the samples were calcined at 925 °C for 28 h in the muffle furnace in air. The calcined samples were ground and sieved up to the fraction of 0.2 mm. The calcines were leached by water at 50–60 °C for 1 h, and then the residue was dried at 300 °C for 2 h. The obtained copper and zinc-copper ferrites were also tested for impurities by XRD analysis.

The ZPR sample was delivered from JSC Chelyabinsk Zinc Plant (Chelyabinsk, Russia, 55.210339° N, 61.376907° E), which currently utilizes ZPR by the Waelz process. The elemental composition of ZPR was analyzed by X-ray fluorescence spectrometer Axios Advanced (PANalytical, Almelo, The Netherlands). The carbon content was determined by CS–400 analyzer (LECO, St. Joseph, MI, USA). Gallium, indium and thallium contents were analyzed by the method of inductively coupled plasma mass spectrometry (ICP-MS) using Elan 6100 DRC (PerkinElmer Inc., Waltham, MA, USA) device. The XRD pattern of ZPR, as well as roasted and leached samples, were obtained by diffractometer ARL X’TRA (Thermo Fisher Scientific, Waltham, MA, USA) using Cu-K_ɑ_ radiation. The qualitative phase analysis was performed by Match! 3.11 Software (Crystal Impact, Bonn, Germany) [51] using Crystallography Open Database. The quantitative phase analysis of zinc, copper and cadmium minerals was carried out using methods of chemical phase analysis [49]; Appendix A presents these methods. The analysis of iron-containing components of ZPR, as well as the roasted and leached samples, was carried out at both 77.5 ± 0.3 K and 296 ± 3 K by Mössbauer spectrometer MS1104EM (CJSC Cordon, Rostov-on-Don, Russia) using the ^57^Co source in rhodium matrix with 16 mCi activity. Mössbauer spectra was processed by SpectrRelax 2.8 software (Lomonosov MSU, Moscow, Russia). The values of chemical shift were presented relative to *α*-Fe.

### 2.2. Thermodynamic Calculation

To simulate equilibrium states at roasting conditions, HSC Chemistry 9.9 software (Outotec, Pori, Finland) [52] was used. Equilibrium compositions were calculated at 300–900 °C using 100 kg of ZPR at P = 0.1 MPa and model gaseous phase containing 79 at. % N_2_ and 21 at. % O_2_. The ZPR composition was calculated according to the results of elemental, XRD and chemical phase analysis. Components of insignificant contents, as well as missing or doubtful thermodynamic data, were neglected from the calculation. Table 1 lists the model composition for the thermodynamic simulation. 

### 2.3. Experimental Procedure

The laboratory experiments were carried out using the mixtures of iron sulfates with either pure ferrites or ZPR. The mixtures in selected ratios were prepared in the V-shell blender and briquetted into tablets of 1 g in molds of 17 mm in diameter by hand hydraulic press at pressure of 40 MPa. Then 3 g of the samples as tablets in a corundum crucible 32 mm high and 25 mm in diameter were placed in a muffle furnace heated to a certain temperature in the range from 575 to 675 °C. The holding time was in the range from 5 to 240 min, then the samples were taken out; after cooling down they were ground and sieved up to the fraction of 0.054 mm.

The roasted ZPR samples were leached by distilled water using a magnetic stirrer at 70 °C with solid to liquid ratio of 1:160 for 30 min. The content of zinc, copper, iron, cadmium, indium, gallium and thallium in the leaching solutions were analyzed by ICP-MS method using Elan 6100 DRC (PerkinElmer Inc., Waltham, MA, USA) device and by atomic emission spectroscopy (AES) method using Varian Vista Pro (Varian Optical Spectroscopy Instr., Mulgrave, Australia) device.

Mechanism of the interactions of ferrites with sulfates was investigated by the methods of synchronous thermal analysis (STA) and time-resolved X-ray diffraction (TRXRD). STA was carried out using simultaneous thermogravimetric and differential scanning calorimetric methods by SDT Q–600 (TA Instruments, New Castle, DE, USA) device. The mixtures of 15–40 mg in weight were heated with a rate of 10 °C/min within the temperature range of 25–900 °C in corundum crucibles in air. TRXRD method can collect XRD data in-situ during roasting [53]. To obtain XRD patterns, the integrated device was used based on DRON (JSC Bourevestnik, Saint-Petersburg, Russia) diffractometer with iron anode and a resistance furnace. The samples were heated up to 625 °C with a rate of 10 °C/min and were held during 15 min. The experiments were carried out in air. Temperature was set by automatic controller using chromel-alumel K-type thermocouple. XRD patterns at 2-theta range 25°–57° were recorded continuously during the heating. The exposure time of an individual XRD pattern was 6 s.

## 3. Results

### 3.1. ZPR Characterization

**Figure 1:**
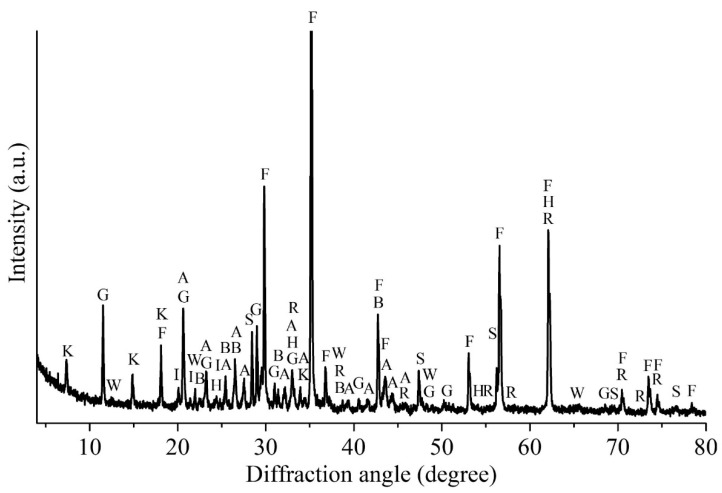
The XRD pattern of the ZPR sample, where F—ZnFe_2_O_4_ (96–151–3088); K—Zn_2_(Cu_4.8_Zn_3.2_)(SO_4_)_4_(OH)_12_·12H_2_O (96–900–8271); G—CaSO_4_·2H_2_O (96–230–0259); A—PbSO_4_ (96–152–8838); I—ZnSO_4_·6H_2_O (96–901–4481); S—Zn_0,9_Fe_0.11_S_0.99_ (96–901–6494); R—Ca_3_Al_2_(SiO_4_)_3_ (96–900–0442); H—α–Fe_2_O_3_ (hematite) (96–901–5066); B—BaSO_4_ (96–900–4486); W—Zn_2_SiO_4_ (96–900–9628).

Table 2 and Table 3 show the elemental composition and the phase distribution of zinc, copper and cadmium in ZPR, respectively. Table 4 demonstrates a water-soluble part of minor valuable elements of ZPR. Figure 1 illustrates the XRD pattern of ZPR. 

The analyses have pointed out that ZPR is a multi-component complex material that contains not only minerals formed during the oxidizing roasting of zinc concentrates such as various ferrites and silicates, but different sulfates formed during the sulfuric acid leaching including water-soluble ones. Zinc, copper and cadmium in ZPR are mostly in complex ferrites, but there are also significant contents of sulfates, oxides, and sulfides unoxidized in fluidized-bed furnace. A water-soluble part of minor elements is proved to be insignificant.

**Figure 2:**
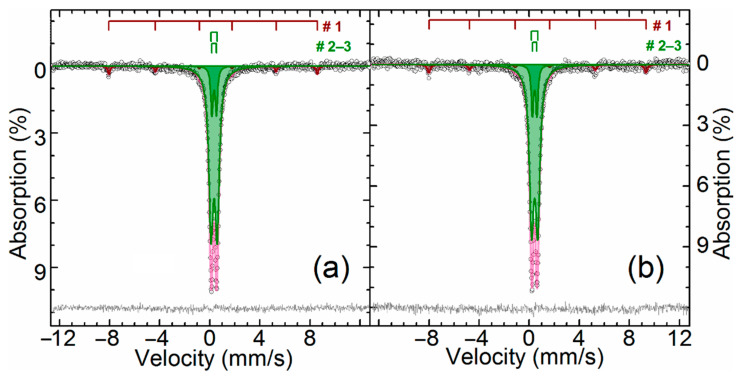
The Mössbauer spectra of the ZPR sample obtained at 296 K (**a**) and 78 K (**b**).

Figure 2 and Table 5 show the results of Mössbauer analysis of iron-containing phases in ZPR.

As can be seen, there is an intense paramagnetic doublet and a very weak sextet on the spectra. The central doublet can be described as a combination of two paramagnetic subspectra with the same isomer shift value of Fe^+3^ atoms in an octahedral oxygen surrounding [54]. The values of isomer shift and quadrupole splitting point out that the doublet is zinc ferrite, which is ferrimagnetic [55] with Neel point about 9 K [56]. Particle size reduction of zinc ferrite can lead to the spectrum distortion already at 77 K [57] owing to increasing Neel point up to 30 K [58], but there is no the spectra distortion, so zinc ferrite, especially related to the inner doublet, is well crystallized. Apparently, the outer doublet corresponds to substituted zinc ferrites Zn_1__−x_A_x_Fe_2__−y_B_y_O_4_, where A and B are substituents with oxidation state +2 or +3, respectively, e.g., Cd^+2^ [59], Mn^+2^ [60], Al^+3^ [61,62], etc. Probably, the substitution of zinc or iron atoms in their crystal sublattices causes an increase in the quadrupole splitting and line width for the outer doublet due to the variation of elements A and B and the degree of substitution x and y.

Low-intensity sextet is hematite [63] due to a specific temperature dependence of the quadrupole shift of α-Fe_2_O_3_ caused by the Morrin transition [64]. Obviously, hematite in ZPR appears during oxidation of iron sulfides during the roasting in fluidized-bed furnace. 

### 3.2. Sulfating of Pure Ferrites

#### 3.2.1. STA Analysis

**Figure 3:**
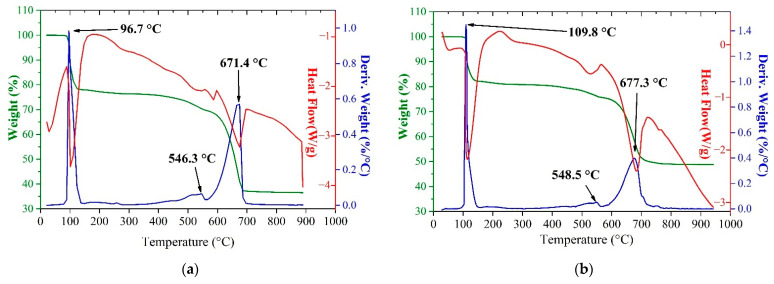
TG-DSC thermogram of pure FeSO_4_∙7H_2_O (**a**) and ZnFe_2_O_4_ + FeSO_4_∙7H_2_O mixture in 1:4 molar ratio excluding hydrated moisture (**b**).

Figure 3 compares TG-DSC curves of pure iron sulfate (II) heptahydrate and ZnFe_2_O_4_ + FeSO_4_∙7H_2_O mixture.

As can be seen, the temperature ranges of all peaks obtained during the heating the mixture of ZnFe_2_O_4_ + FeSO_4_∙7H_2_O approximately match with the temperature ranges of peaks of thermal decomposition of pure FeSO_4_∙7H_2_O. All obtained thermograms for the ferrite–sulfate systems involving ZnFe_2_O_4_, CuFe_2_O_4_, Zn_0.5_Cu_0.5_Fe_2_O_4_, FeSO_4_∙7H_2_O, Fe_2_(SO_4_)_3_∙9H_2_O were similar in the temperature ranges of peaks to the thermal decomposition of iron sulfates (see dataset [65]). It indicates the identity of the interaction mechanism in all the studied ferrite–sulfate systems. Evidently, reactions of the ferrites with the sulfates occur in the range of 550–700 °C that coincide with sulfur oxides removal.

#### 3.2.2. Roasting Experiments

Firstly, to verify the ferrite–sulfate reactions, roasting experiments with pure compounds followed by XRD analysis of the roasted samples were carried out at 550–700 °C. Table 6 shows the general results of the experiments for all the systems. As in the case of STA analysis similarity, the XRD patterns of the roasted samples at the same temperature were qualitatively identical for all the ferrite–sulfate systems.

It is clear that across the entire temperature range the ferrites decomposes to form zinc and copper sulfates. The sulfating degree was significant in the samples obtained at 600 and 650 °C, while it was less 5% in the samples obtained at 550 and 700 °C. However, it should be noted that besides Fe_2_O_3_, which is the main phase of all the obtained samples, the amount of undecomposed ferrites was also substantial at all the temperatures and ratios. Based on XRD analysis, quantitative dependences of the sulfating degree on temperature, roasting time and mixture ratios in the experiments with pure compounds is proved to be equivocal.

#### 3.2.3. TRXRD Experiments

**Figure 4:**
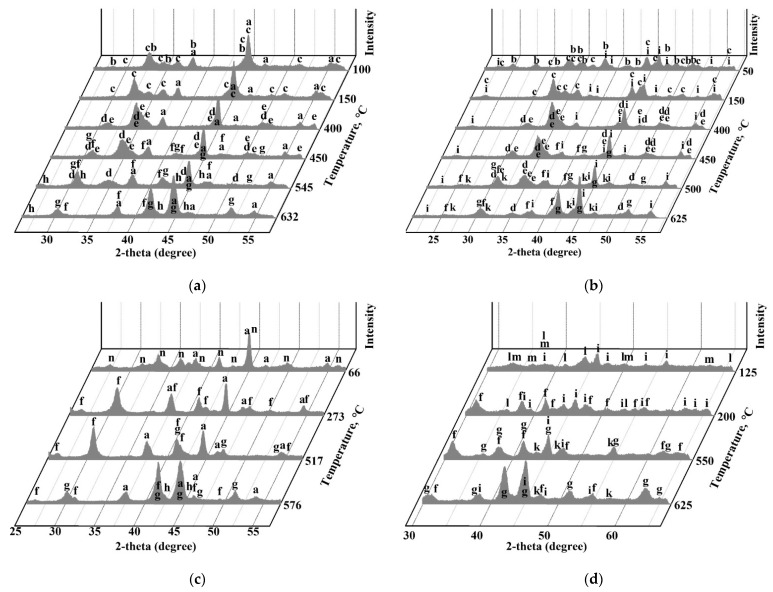
The XRD patterns obtained at different temperatures during TRXRD experiments using mixtures of 18.2% ZnFe_2_O_4_ + 81.8% FeSO_4_∙7H_2_O (**a**), 18.1% CuFe_2_O_4_ + 81.9% FeSO_4_∙7H_2_O (**b**), 17.2% ZnFe_2_O_4_ + 82.8% FeSO_4_∙7H_2_O (**c**) and 17.1% CuFe_2_O_4_ + 82.9% FeSO_4_∙7H_2_O (**d**), where a—ZnFe_2_O_4_; b—FeSO_4_∙4H_2_O; c—FeSO_4_∙H_2_O; d—FeSO_4_; e—Fe(OH)SO_4_; f—Fe_2_(SO_4_)_3_; g—Fe_2_O_3_; h—ZnSO_4_; i—CuFe_2_O_4_; k—CuSO_4_; l—Fe_2_(SO_4_)_3_∙5H_2_O; m—Fe_2_(SO_4_)_3_∙7H_2_O; n—Fe_2_(SO_4_)_3_∙9H_2_O.

To find out the reactions in the systems, TRXRD experiments were carried out. Figure 4 shows the XRD patterns at different temperatures during heating of the mixtures of FeSO_4_∙7H_2_O and Fe_2_(SO_4_)_3_∙9H_2_O with zinc and copper ferrites.

It was revealed that FeSO_4_∙7H_2_O decomposes and transforms sequentially with temperature increasing to trivalent anhydrous sulfate according to the scheme:

FeSO_4_ FeSO_4_∙7H_2_O → FeSO_4_∙4H_2_O → FeSO_4_∙H_2_O → Fe(OH)SO_4_ → Fe_2_(SO_4_)_3_ Fe_2_O(SO_4_)_2_ (1)

These results are generally consistent with [66,67]. Fe_2_(SO_4_)_3_∙9H_2_O also dehydrates sequentially according to the scheme:

Fe_2_(SO_4_)_3_∙9H_2_O → Fe_2_(SO_4_)_3_∙7H_2_O → Fe_2_(SO_4_)_3_∙5H_2_O → Fe_2_(SO_4_)_3_ (2)

Then Fe_2_(SO_4_)_3_ transforms into hematite, and zinc or copper sulfates appear simultaneously. Thus, the performed investigation has found out the mechanism of the sulfating process. Above 500 °C decomposition of Fe_2_(SO_4_)_3_ and sulfating of ferrites by emitted sulfur trioxide occur according to the reactions:

Fe_2_(SO_4_)_3(s)_ → Fe_2_O_3(s)_ + 3SO_3(g)_ (3)

ZnFe_2_O_4(s)_ + SO_3(g)_ → ZnSO_4(s)_ + Fe_2_O_3(s)_ (4)

CuFe_2_O_4(s)_ + SO_3(g)_ → CuSO_4(s)_ + Fe_2_O_3(s)_ (5)

As a generalization, both FeSO_4_∙7H_2_O and Fe_2_(SO_4_)_3_∙9H_2_O affect similarly on the sulfation of ferrites that agrees well with another study [68].

**Figure 5:**
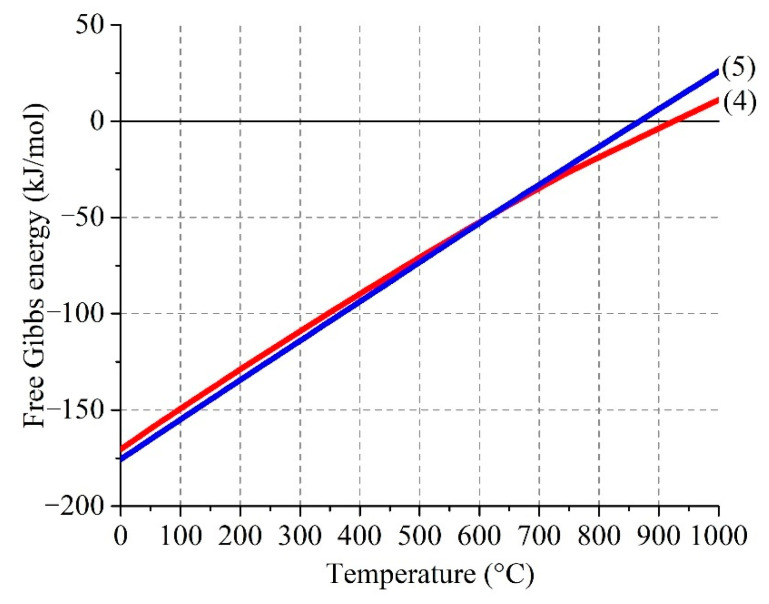
Effect of temperature on Gibbs free energy of the interactions of sulfur trioxide with zinc (4) and copper (5) ferrites.

Figure 5 demonstrates the temperature dependence of Gibbs free energy of the reactions (4) and (5).

As reflected by the plot, the reactions (4) and (5) are possible up to 920 °C and 860 °C, respectively. The interval of existence of SO_3(g)_ in the ferrite–sulfate systems depends on the reaction (3), as well as following reaction in the gas phase:

SO_2(g)_ + 0.5O_2(g)_ ↔ SO_3(g)_ (6)

**Figure 6:**
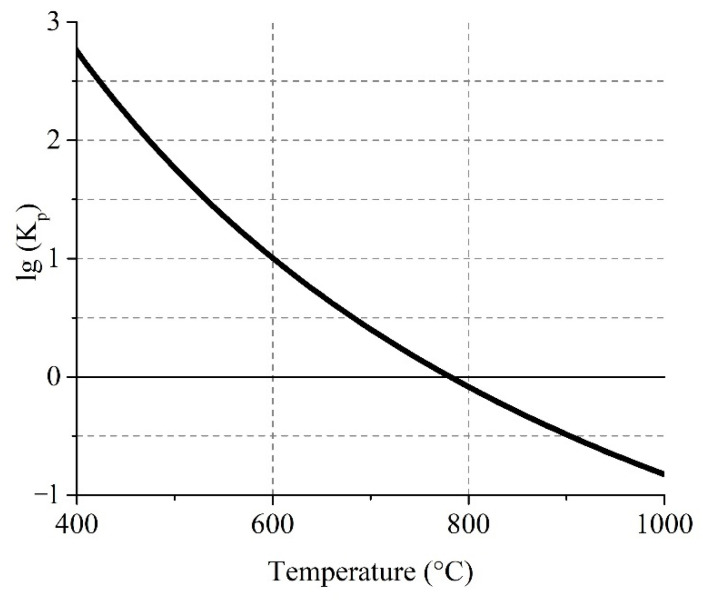
Effect of temperature on the logarithm of the equilibrium constant of the reversible reaction (6).

Figure 6 shows the temperature dependence of Gibbs free energy for the gas reaction (6) of sulfur trioxide generation.

The equilibrium curve evidences that forward reaction (6) of SO_3(g)_ formation is possible at temperatures up to 780 °C. The logarithm of the equilibrium constant of the reaction at 680 °C and lower have a value above 0.5 with a significant proportion of SO_3(g)_. This fact explains the obtaining of inconsiderable sulfation degrees of pure ferrites at 700 °C (Section 3.2.2). However, it should be noted that at 550 °C there was also an unessential sulfation degree that is probably due to an insignificant decomposition rate of iron sulfate at such conditions according to the reaction (3).

### 3.3. Sulfating of ZPR

#### 3.3.1. Thermodynamic Calculation

Based on the results of the experiments with pure ferrites, the temperature ranges from 575 to 675 °C was chosen for the sulfating of ZPR. Due to the similarity of the effect of both iron sulfates during roasting, only Fe_2_(SO_4_)_3_∙9H_2_O was used in the experiments with ZPR.

**Figure 7:**
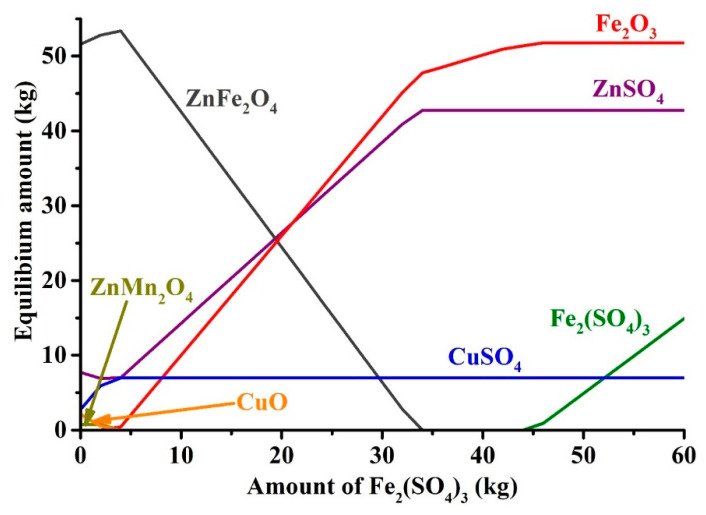
Effect of Fe_2_(SO_4_)_3_ addition to 100 kg of ZPR on the equilibrium amounts of Zn, Cu and Fe compounds at 625 °C.

To determine the required amount of iron (III) sulfate for zinc and copper sulfation, the thermodynamic simulation was carried out. Figure 7 points out an influence of the addition of anhydrous iron (III) sulfate on equilibrium amounts of zinc, copper and iron compounds in ZPR at the roasting conditions.

As can be seen, to convert zinc and copper ferrites and other compounds into the sulfates, approximately 34 kg Fe_2_(SO_4_)_3_ should be added to 100 kg of ZPR that is equivalent to 48 kg Fe_2_(SO_4_)_3_∙9H_2_O. The calculated amount of the sulfating agent was used during subsequent experiments.

#### 3.3.2. Influence of Roasting Temperature on the Behavior of Valuable Elements in ZPR

**Figure 8:**
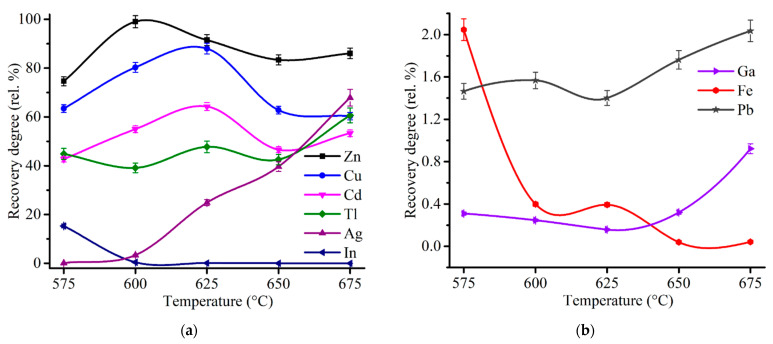
Effect of the roasting temperature on the recovery degree of Zn, Cu, Cd, In, Tl, Ag (**a**), as well as Ga, Fe and Pb (**b**) after 180 min of the roasting and subsequent water leaching with the addition of 48% Fe_2_(SO_4_)_3_·9H_2_O to 100% ZPR.

Firstly, an effect of the roasting temperature was investigated at 180 min of the duration. Figure 8 demonstrates an influence of the temperature on the recovery degree of the metals after the roasting and subsequent water leaching.

The recovery rate of zinc reaches 99% at 600 °C and then gradually decreases with the temperature increase. Therefore, it can be stated clearly that sulfating of zinc minerals in ZPR, namely, zinc ferrite, zinc oxide, zinc sulfide and zinc silicate, are possible almost completely according to the reaction (4), as well as the following reactions [42]:

ZnS_(s)_ + 2O_2(g)_ = ZnSO_4(s)_ (7)

ZnO_(s)_ + SO_3(g)_ = ZnSO_4(s)_ (8)

Zn_2_SiO_4(s)_ + 2SO_3(g)_ = 2ZnSO_4(s)_ + SiO_2(s)_ (9)

**Figure 9:**
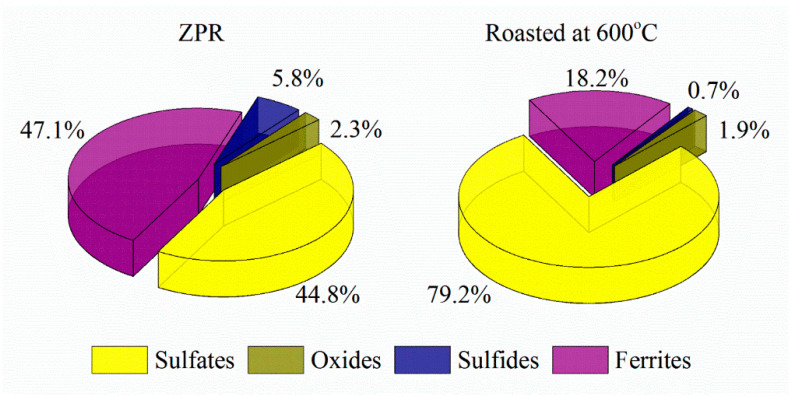
Copper phase distribution in ZPR and in the roasted sample obtained at 600 °C after 180 min of the roasting with the addition of 48% Fe_2_(SO_4_)_3_·9H_2_O to 100% ZPR.

**Figure A4:**
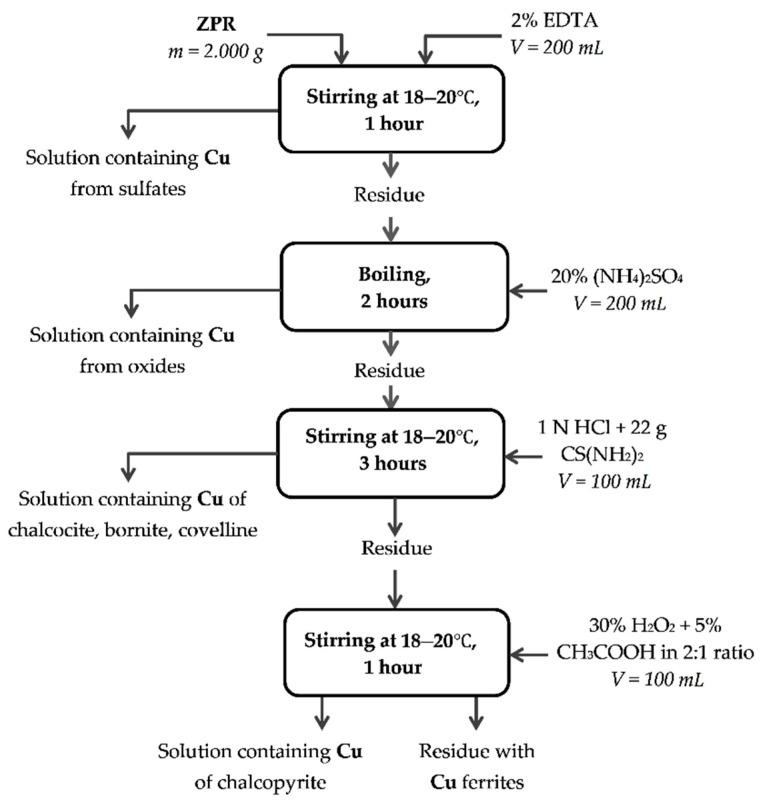
Method of chemical phase analysis of copper compounds.

The recovery degree of copper proved to be considerable but incomplete. At 600 and 625 °C copper recovery rate was 80.3% and 88.0%, respectively. To determine unambiguously the remaining phases that are present in the residue after the roasting at 600 °C, chemical phase analysis of copper phases in the roasted sample was carried out according to the scheme illustrated in Figure A4. Figure 9 compares copper phase distribution in ZPR and in the roasted ZPR sample.

As is clear from the pie chart, a significant part of Cu remains in the roasted sample as a ferrite. Probably, sulfating of copper phases is similar to zinc, but despite the thermodynamic possibility, reaction (5) has a hindrance during the roasting at these conditions. The experiments have shown (Figure 8) that although the roasting at 625 °C leads to increasing of copper recovery degree compared with 600 °C, the sulfating degree of copper ferrite are still less fully than the sulfating degree of zinc ferrite. However, the obtained copper recovery degrees of about 80% is quite satisfactory taking into account the extraction of 99% Zn.

Cadmium recovery rate is qualitatively similar the copper curve and ranges from 43 to 64%. As shown in Table 3, the main part of cadmium in ZPR is in the ferrite or other low-soluble forms. It leads to the assumption that as for copper ferrites, the sulfating of cadmium ferrites proceeds incompletely in the temperature range under study.

The solubility of lead in water is insignificant due to the presence of the main part of Pb in the sulfate form both before the roasting (Figure 1) and obviously after it.

Silver recovery degree increases with a temperature rise that is likely due to a promotion by the roasting temperature of the reaction of silver sulfating:

2Ag_(s)_ + SO_2(g)_ + O_2(g)_ = Ag_2_SO_4(s)_ (10)

As silver sulfate solubility in water is substantial [69], it is expedient to suppress its formation to remain Ag almost completely in the residue. As can be seen from Figure 8a, the roasting temperature of 600 °C is quite suitable for this purpose.

As can be expected, the main part of iron is insoluble owing to decomposition of unreacted ferric sulfate according to the reaction (3). The dissolution degree of iron dramatically drops with an increase of the roasting temperature from 575 to 675 °C. High separation efficiency of zinc and iron should be noted.

The recovery degree of indium and gallium are less than 1% across the entire temperature range except for 575 °C with 15% In passed into solution. The remaining of In and Ga in the residue under the roasting conditions is likely due to the decomposition of the water-soluble sulfates to the corresponding oxides of low solubility [70] at lower temperatures according to the reactions [71,72]:

In_2_(SO_4_)_3(s)_ = In_2_O_3(s)_ + SO_3(g)_ (11)

Ga_2_(SO_4_)_3(s)_ = Ga_2_O_3(s)_ + SO_3(g)_ (12)

The recovery rate of thallium is in the range from 40 to 60%, which is considerably different compared with the indium and gallium recovery rate because water-soluble Tl_2_SO_4_ is stable within the temperature range of the roasting [73].

Thus, the sulfating roasting at 600 °C and following water leaching lead to the recovery degree of Zn and Cu of 99.0% and 80.3%, respectively, and almost full remaining of Fe, Pb, Ag, In, Ga in the residue.

#### 3.3.3. Influence of Iron Sulfate Amount on the Behavior of Valuable Elements in ZPR

**Figure 10:**
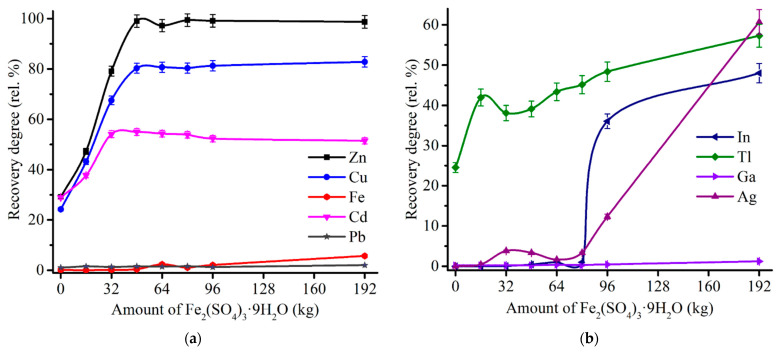
Effect of Fe_2_(SO_4_)_3_∙9H_2_O addition to 100% ZPR on the recovery degree of Zn, Cu, Cd, Fe, Pb (**a**), as well as In, Tl, Ga, Ag (**b**) after 180 min of the roasting at 600 °C and subsequent water leaching.

Effect of the sulfating agent amount was investigated at 600 °C of the roasting temperature and 180 min of the duration. Figure 10 illustrates an influence of Fe_2_(SO_4_)_3_∙9H_2_O addition to ZPR on the recovery rate of major and minor elements after following water leaching.

As shown in Figure 10a, the recovery rates of zinc, copper and cadmium achieve plateau in the ranges of 97–99% Zn, 80–83% Cu, 51–55% Cd with the addition over 48% Fe_2_(SO_4_)_3_∙9H_2_O that is consistent with the results of thermodynamic simulation (Figure 7). As anticipated, an increment of the sulfating agent addition causes an increase of iron dissolution degree due to a probably higher amount of undecomposed iron sulfate in the roasted samples. Moreover, an increased consumption of Fe_2_(SO_4_)_3_∙9H_2_O not only is cost-inefficient, but leads to a significant rise of dissolution degree of In and Ag (Figure 10b) that is unfavorable for their selective extraction.

#### 3.3.4. Kinetics of ZPR Sulfating Roasting

**Figure 11:**
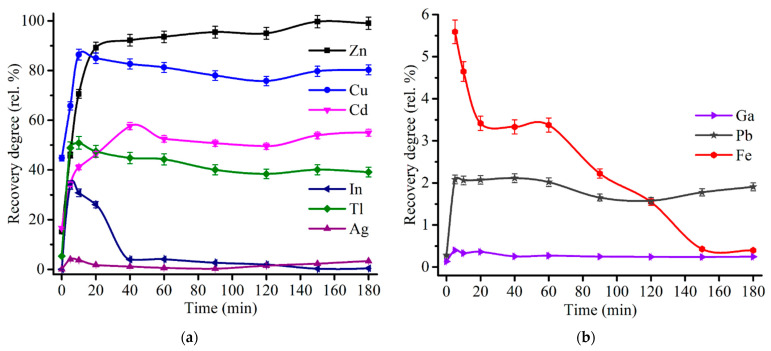
Effect of roasting time on the recovery degree of Zn, Cu, Cd, In, Tl, Ag (**a**), as well as Ga, Fe and Pb (**b**) at 600 °C with the addition of 48% Fe_2_(SO_4_)_3_·9H_2_O to 100% ZPR and subsequent water leaching.

Figure 11 shows an influence of the roasting time on the recovery degree of major and minor elements at 600 °C and 48% Fe_2_(SO_4_)_3_∙9H_2_O addition to ZPR. As can be seen from the plots, the sulfating of zinc, copper, cadmium and thallium is quite rapid process, which occurs almost fully in the range from 5 to 40 min. Furthermore, the same roasting duration ranges enable to suppress the dissolution of indium and gallium. In contrast with the other elements, the dissolution degree of iron achieves a plateau only after 150 min of the roasting at a minimum level of 0.4%.

**Figure 12:**
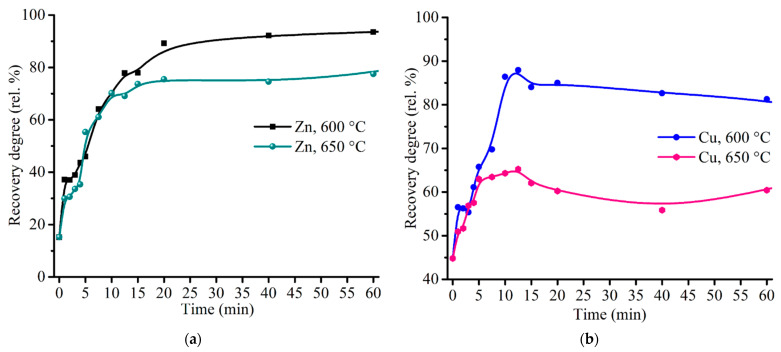
Effect of roasting time on the recovery degree of zinc (**a**) and copper (**b**) at 600 and 650 °C with the addition of 48% Fe_2_(SO_4_)_3_·9H_2_O to 100% ZPR and subsequent water leaching.

To elucidate a behavior of zinc and copper during the sulfating process, the additional experiments were carried out at the roasting time up to 20 min. Figure 12 gives kinetic curves of zinc and copper sulfation. It should be noted from Figure 11a and Figure 12b that copper achieved a maximum recovery degree at 10.0–12.5 min, then a sloping decreasing occurs. Such copper behavior is likely due to a partial decomposition of copper sulfate to oxide and subsequent ferritization, so the elevated temperature and the prolonged roasting duration are unfavorable for copper recovery.

Experimental data were processed by reduced time method [74] using various well-known kinetic models. The zinc kinetic curve was divided into two sections of high and low rate, namely, from 0 to 12.5 min and from 12.5 to 90 min, respectively. The copper kinetic curve was processed only for the section of high rate from 0 to 12.5 min due to a peculiar behavior of copper at longer duration described above. Table 7 lists the results of the data processing and indicates adequacy of the models for the experimental data.

As follows from the kinetic calculation, the initial section of the zinc and copper curves fits Erofeev–Avrami equation with *n* = 2, which points out that generation and growth of nuclei is a rate-controlling step in the range from 0 to 12.5 min. However, it should be noted that the models of shrinking sphere and chemical reaction of first order are also quite suitable for the experimental data due to high values of adjusted R-squared. Therefore, one cannot exclude a mixed mode that can consist of two rate-controlling steps, namely, nuclei generation and growth, as well as chemical process, e.g., the reaction (3). The second section of the reaction for zinc corresponds well to Erofeev–Avrami equation with *n* = 3, while the reaction-controlled equations are unfit, so rate-controlling by nuclei generation and growth in the range from 12.5 to 90 min is a fair assumption.

#### 3.3.5. Characterization of Roasted and Water-Leached ZPR

**Figure 13:**
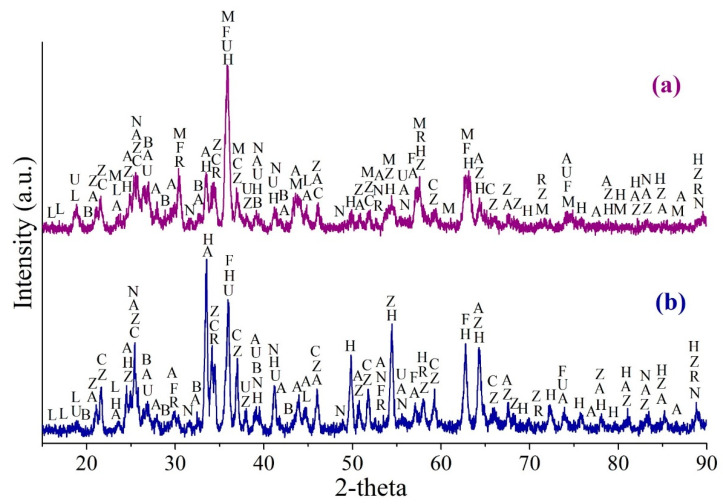
The XRD pattern of the ZPR sample roasted with the addition of 48% Fe_2_(SO_4_)_3_∙9H_2_O during 180 min at 600 (**a**) and 625 °C (**b**), where H—α-Fe_2_O_3_ (hematite) (96–901–4881); M—γ-Fe_2_O_3_ (maghemite) (96–152–8613); F—ZnFe_2_O_4_ (96–151–3088); A—PbSO_4_ (96–900–0653); U—ZnSO_4_·H_2_O (96–900–9374); Z—ZnSO_4_ (96–900–7445); L—CuSO_4_∙5H_2_O (96–101–0528); C—CuSO_4_ (96–231–0621); R—Ca_3_Al_2_(SiO_4_)_3_ (96–900–2680); N—CaSO_4_ (96–500–0041); B—BaSO_4_ (96–100–0038).

Figure 13 demonstrates the XRD patterns of roasted ZPR at 600 °C and 625 °C. Although the roasting conditions is almost similar with distinction of the temperature of only 25 °C, the XRD patterns has a substantial difference. The main iron-containing phase of the sample roasted at 600 °C is maghemite (γ-Fe_2_O_3_); moderate peaks of hematite (α-Fe_2_O_3_) and low-intensity peaks of the remaining ferrite are also present (Figure 13a). The sample roasted at 625 °C contains most of iron in hematite, the ferrite appears to have insignificant but higher intensive peaks than in the other sample that is consistent with the results of the experiments illustrated in Figure 8a. Maghemite in a substantial proportion is proved to be undetected in the sample roasted at 625 °C (Figure 13b). Other phases such as anhydrous and hydrated zinc and copper sulfates, as well as containing in the original ZPR grossular and sulfates of lead, calcium and barium are qualitatively similar in both the samples.

**Figure 14:**
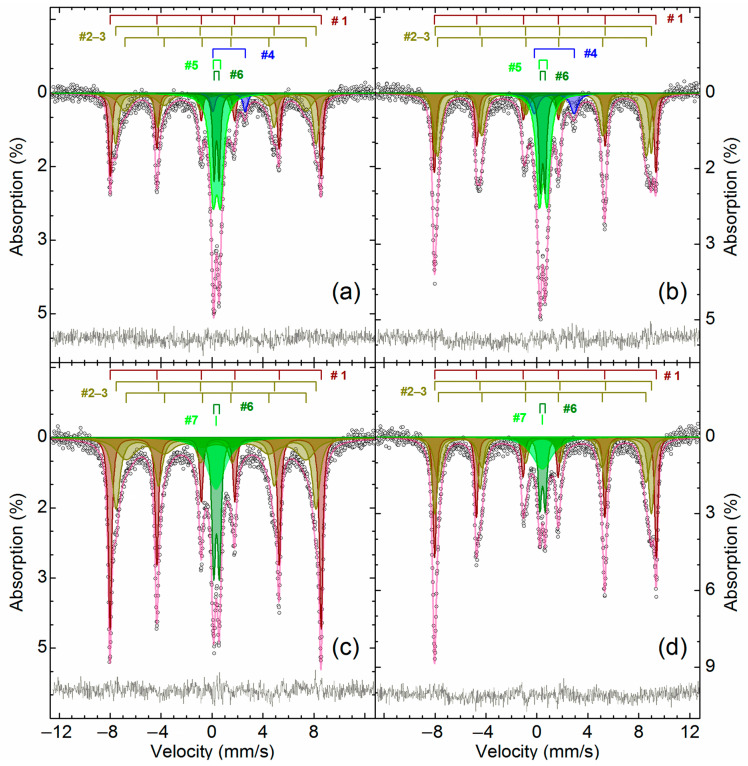
The Mössbauer spectra obtained at 296 K (**a**,**c**) and 78 K (**b**,**d**) of ZPR roasted with the addition of 48% Fe_2_(SO_4_)_3_∙9H_2_O during 180 min at 600 °C (**a**,**b**), as well as of the same sample after its water leaching (**c**,**d**).

To elucidate iron distribution in the maghemite-containing sample, Mössbauer analysis of the sample roasted at 600 °C before and after water leaching was carried out. Figure 14 illustrates the Mössbauer spectra of the roasted and leached samples, Table 8 gives the parameters of the illustrated spectra. Appendix B includes the parameters of the supplementary samples, which provided to clarify and confirm our hypotheses regarding phase identification.

The obtained Mössbauer spectra has an appreciable temperature dependence. The temperature decrease of the spectra collecting causes a narrowing and significant intensity increasing of lines 1 and 5, as well as broadening of lines 2 and 6 that indicates the presence of a superposition of several sextets. Three sextets correspond to Fe^+3^ atoms in an octahedral oxygen environment, but belong to different phases. The parameters of sextet #1 is similar to the sextet detected in the sample of the original ZPR (Figure 14 and Table 8) and corresponds to α-Fe_2_O_3_ [75]. Evidently, two other sextets (##3–4, Table 8) are related to γ-Fe_2_O_3_ [76,77,78]. The reduced values of the magnetic splitting for the sextets at room temperature and the high value of the resonance line width with a strong temperature dependence suggest that this is a poorly ordered and very defective phase [79]. It could be assumed that the group of the sextets ##1–3 also includes the group of copper ferrite sextets with ultrafine parameters close to the indicated oxides—CuFe_2_O_4_ [80,81,82,83] or substituted analogs of copper ferrite Cu_1-x_A_x_Fe_2-y_B_y_O_4_, where A and B are a substituent element in the oxidation state +2 or +3, respectively, for example, Zn^+2^ [84,85,86], Cd^+2^ [85], Al^+3^ [87], Ga^+3^ [86]. However, this hypothesis was not confirmed because after calcination of these samples at 900 °C, no other iron-containing phases were found in the samples except for zinc ferrite and α-Fe_2_O_3_ (Appendix B).

**Figure 15:**
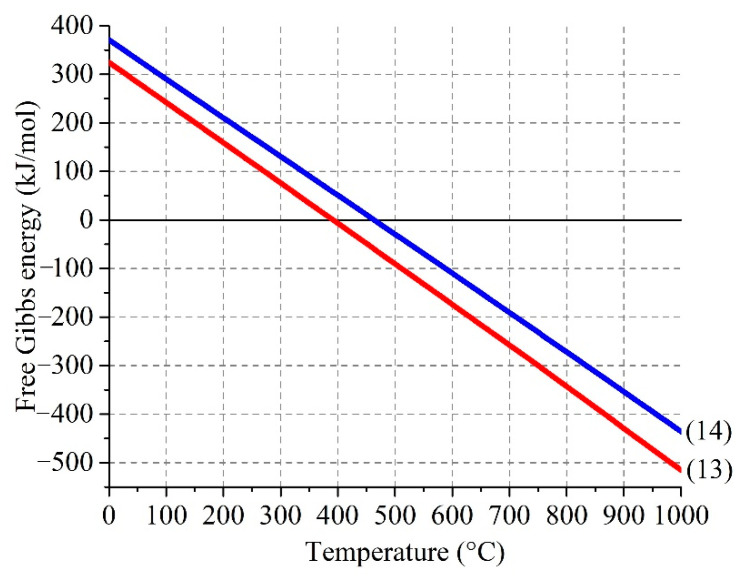
Effect of temperature on Gibbs free energy of the interactions of ferric sulfate with zinc (4) and copper (5) sulfides.

The doublets ##4–5 (Figure 14 and Table 8), which is present in the roasted sample, but is absent in the water-leached sample, corresponds to Fe^+2^ and Fe^+3^ atoms in an octahedral oxygen environment [54], respectively. The hyperfine parameters of #4 for both the temperatures are in good agreement with the data for FeSO_4_∙H_2_O [88,89,90]. The presence of Fe^+2^ is inconsistent with the results of the Section 3.2, so it is an open question how FeSO_4_∙H_2_O was formed during the roasting. As shown in Figure 15, it is thermodynamically possible that divalent iron can be a product of interactions of Fe_2_(SO_4_)_3_ with sulfides containing in a substantial amount (Table 3) in ZPR according to the reactions:

4Fe_2_(SO_4_)_3(s)_ + ZnS_(s)_ = 8FeSO_4(s)_ + ZnSO_4(s)_ + 4SO_3(g)_ (13)

5Fe_2_(SO_4_)_3(s)_ + Cu_2_S_(s)_ = 10FeSO_4(s)_ + 2CuSO_4(s)_ + 4SO_3(g)_ (14)

However, this fact, as well as the fact of the presence of γ-Fe_2_O_3_, should be investigated further.

Doublet #5 obviously corresponds to the iron (III) sulfate dehydration products [91,92,93], e.g., Fe_2_O(SO_4_)_2_∙xH_2_O [66]. The very broadened singlet #7 in the water-leached sample can be related to Fe^+3^ atoms in an octahedral oxygen environment [52] and, apparently, corresponds to amorphous products of hydrolysis of soluble iron salts. After the additional calcination of the samples at 900 °C, all the indicated paramagnetic iron-containing compounds are not detected (Appendix B). The doublet #6, which has the same parameters with the one in the ZPR sample, is unsulfated zinc ferrite.

Thus, the characterization of the roasted and water-leached samples has pointed out that the sample treated under the optimal conditions to obtain the highest recovery degree of valuable elements contains the main part of iron in the form of γ-Fe_2_O_3_ rather than α-Fe_2_O_3_. Taking this result into account, it is possible to apply low-intensity magnetic separation to extract iron from the residue rather than high-intensity magnetic separation used in [40] to recover α-Fe_2_O_3_ that can improve the efficiency of iron separation.

## 4. Discussion

The investigation on the mechanism of sulfating roasting using pure zinc and copper ferrites has pointed out that the process occurs through an interaction of the ferrites with SO_3(g)_, which are generated through decomposition of iron sulfates, to form appropriate sulfates. Therefore, it can be inferred that the reversible gas-phase reaction (6) is a crucial process to control efficiency of sulfation of the ferrites. These results are consistent with [68], where a similar conclusion was drawn, but inconsistent with [40], where authors claimed that zinc ferrite transforms into sulfate via interaction with SO_2(g)_. However, it should be noted that authors of [40] used gas mixtures of sulfur dioxide and air. This leads to the assumption that, as previously stated, sulfur trioxide generated during the reversible reaction (6) play a key role during the gaseous sulfating of the ferrites.

It is deduced, based on the previous discussion, that sulfation process can be realized using both gaseous and solid sulfating agents. Although gaseous sulfation seems to be more promising due to a possible variation of the roasting temperature in a wider range than with the application of solid sulfating agents, authors [41] reported that industrial implementation of gaseous sulfation in a conventional fluidized bed reactor is impracticable because of sticking of formed sulfates to the reactor walls causing the maintenance problems. Moreover, gaseous sulfation requires a proper charge preparation and a binder addition for efficient operation in a selected metallurgical unit. Therefore, to avoid the problems, the gaseous sulfation requires suitable metallurgical units to prevent the undesirable effects that restricts an industrial application of the gaseous sulfation method.

The sulfation roasting using iron sulfates have some advantages over gaseous sulfation such as lack of binders due to the binding property of iron sulfates [94,95,96], the possibility of the application of conventional metallurgical furnaces for the roasting. Our study has shown that there is no difference in the mechanism of the both iron (II) and (III) sulfates influence; the distinction is only a different required amount of a sulfating agent based on the one or the other iron sulfate for the process. In addition, it is well-known that the reaction (6) is significantly catalyzed by iron oxides [97], which generated through the decomposition of the iron sulfates during the roasting, so the sulfation using iron sulfates is an autocatalytic process that seems to be important factor to obtain such a high zinc recovery degree. Furthermore, it is interesting to note that the best recovery degrees of valuable elements in the experiments using the mixture of ZPR and iron (III) sulfate under optimal conditions were obtained with a simultaneous generation of γ-Fe_2_O_3_ in the roasted sample, while the presence of only α-Fe_2_O_3_ led to lower recovery degrees. However, it was reported that the catalytical effect of γ-Fe_2_O_3_ on the reaction (6) is slightly less than α-Fe_2_O_3_ [98,99], therefore, not only the autocatalysis is the reason of high recovery degrees under optimal conditions.

The sulfating roasting at 600 °C during 180 min with addition of 48% Fe_2_(SO_4_)_3_∙9H_2_O to 100% ZPR and subsequent water leaching led to the recovery of 99.0% Zn, 80.3% Cu, 55.0% Cd, 39.0% Tl into the solution, as well as retaining of 99.6% Fe, 98.1% Pb, 99.6% In, 99.8% Ga, 96.6% Ag in the solid residue. Copper and cadmium can be conventionally extracted and separated from the leached solution by zinc dust cementation [6,100], then the zinc-containing solution after its appropriate purification can be used for zinc electrowinning. Lead, which is in the solid residue as PbSO_4_, can be recovered using leaching by NaOH solution [101,102]. Undoubtedly, whatever method was chosen for iron extraction from the remaining part, economic feasibility of the sequential process can be achieved providing iron regeneration into solid sulfate to recirculate it for the roasting. The same applies to off-gas for the roasting process, which needs to be recycled through a conventional contact method of sulfuric acid production. Economic assessment of the recovery of minor elements such as In, Ga, Tl and Ag from both leached solution and solid residue should be carried out depending on their contents in the original ZPR and difficulty to extract them from intermediate products.

## 5. Conclusions

The study has shown that sulfating of ZnFe_2_O_4_, CuFe_2_O_4_ and Zn_0.5_Cu_0.5_Fe_2_O_4_ by FeSO_4_∙7H_2_O and Fe_2_(SO_4_)_3_∙9H_2_O at the range from 550 to 700 °C is based on the interaction of the ferrites with sulfur trioxide generated through the sulfate decomposition, so the reversible reaction SO_2(g)_ + 0.5O_2(g)_ ↔ SO_3(g)_ is a controlling stage of the sulfating.

ZPR has a complex composition and contains zinc and copper mainly as ferrites, as well as sulfides, sulfates, oxides and silicates. The sulfating roasting under optimal conditions, which are the roasting temperature of 600 °C, roasting duration of 180 min, the addition of 48% Fe_2_(SO_4_)_3_∙9H_2_O to 100% ZPR, and subsequent water leaching led to recovery of 99% Zn and 80.3% Cu, while Fe, Pb, Ag, In, Ga retained almost fully in the residue. Thus, the approach based on the sulfating roasting using iron sulfates has a high efficiency for the extraction of valuable elements from ZPR and can be used as a crucial stage for comprehensive processing of various secondary materials containing zinc in ferrite form.

## Figures and Tables

**Table 1:** Model composition of the ZPR sample for thermodynamic calculation, wt. %.

Species	Content	Species	Content	Species	Content	Species	Content	Species	Content
ZnFe_2_O_4_	40.56	CuFe_2_O_4_	5.13	PbSO_4_	7.99	H_2_O	2.94	Ca_3_Al_2_Si_3_O_12_	4.84
ZnS	1.34	CuSO_4_∙5H_2_O	4.68	BaSO_4_	2.12	CdO	0.38	SiO_2_	1.88
ZnSO_4_∙6H_2_O	10.86	Cu_2_S	0.18	FeS	2.25	CdSO_4_	0.14	Fe_2_O_3_	1.84
ZnO	2.67	CuO	0.08	CaCO_3_	2	CdS	0.05	As_2_O_3_	0.50
Zn_2_SiO_4_	1.09	CaSO_4_∙2H_2_O	4.32	MnS	1.19	MgO	0.95	CuFeS_2_	0.02

**Table 2:** Chemical composition of the ZPR sample ^1^.

Element	Zn	Cu	Cd	Fe	Pb	S	C	Al	Si	Mn	Ca	Mg	Ba	Sb	As	Ag	Ga	In	Tl
Unit	wt.%															mg/kg			
content	17.32	2.66	0.44	23.36	5.46	5.72	0.24	0.58	1.92	0.75	3.10	0.57	1.25	0.07	0.38	283	193	170	20

^1^ the rest are other minor elements and oxygen.

**Table 3:** Phase composition of zinc, copper and cadmium minerals in the ZPR sample.

Phase	Zn Distribution, Rel. %	Phase	Cu Distribution, Rel. %	Phase	Cd Distribution, Rel. %
Zn sulfates	15.2	Cu sulfates	44.8	Cd sulfates	16.6
Zn oxide	12.4	Cu oxides	2.3	Cd oxide + Cd silicates	13.7
Zn silicates + Zn arsenates	3.7	Cu_2_S + CuS + Cu_5_FeS_4_	5.5	Cd sulfides	0.8
Zn sulfides	5.2	CuFeS_2_	0.3	Cd ferrites	37.9
Zn ferrites	63.5	Cu ferrites	47.1	Cd low-soluble	31

**Table 4:** Percentage of water-soluble forms of silver, gallium, indium and thallium in ZPR.

Element	Ag	Ga	In	Tl
solubility in water, rel. %	not detected	0.1	not detected	5.3

**Table 5:** Parameters ^1^ of the Mössbauer spectra of the ZPR sample.

Temperature of Spectra Collecting, K		296					78				
Subspectrum, #	Phase	δ	Δ = 2ε	Γ_exp_	H_eff_	S	δ	Δ = 2ε	Γ_exp_	H_eff_	S
		mm/s	mm/s	mm/s	kOe	%	mm/s	mm/s	mm/s	kOe	%
1	α-Fe_2_O_3_	0.37	-0.21	0.22	516.1	4.5	0.46	0.40	0.27	537.4	5.5
2	Zn_1−x_A_x_Fe_2−y_B_y_O_4_	0.34	0.50	0.43	-	83	0.43	0.50	0.44	-	82
3	ZnFe_2_O_4_	0.34	0.36	0.21	-	13	0.43	0.36	0.21	-	13

^1^ δ—isomer shift; Δ = 2ε—quadrupole splitting; Γ_exp_—line width; H_eff_—hyperfine magnetic field; S—relative area of a subspectrum #.

**Table 6:** General results of XRD analysis of the samples obtained in the experiments in the systems MFe_2_O_4_–FeSO_4_∙7H_2_O and MFe_2_O_4_–Fe_2_(SO_4_)_3_∙9H_2_O, where M is Zn, Cu or Zn_0.5_Cu_0.5_, at 550–700 °C for 60–240 min in molar ratios from 1:0.5 to 1:4 excluding hydrated moisture.

Temperature, °C	Phase Composition of Roasted Samples
550	α-Fe_2_O_3_ MFe_2_O_4_ Fe_2_(SO_4_)_3_ MSO_4_∙nH_2_O (<5%) unidentified phases
600	α-Fe_2_O_3_ M_x_Fe_2_O_4_ MSO_4_∙nH_2_O
650	α-Fe_2_O_3_ M_x_Fe_2_O_4_ MSO_4_∙nH_2_O
700	α-Fe_2_O_3_ M_x_Fe_2_O_4_ MSO_4_∙nH_2_O (<5%)

**Table 7:** Comparison of various kinetic models for the sulfating zinc and copper during the roasting at 600 and 650 °C with the addition of 48% Fe_2_(SO_4_)_3_·9H_2_O to 100% ZPR.

Model Name	Integral Form	Time Range, min	T, °C	R^2^ (Adjusted R-Squared)		T, °C	R^2^ (Adjusted R-Squared)	
				Zn	Cu		Zn	Cu
Jander	[1 − (1 − x)^1/3^]^2^	0–12.5	600	0.7247	0.5797	650	0.8275	0.7160
		12.5–90	600	0.3372	-	650	0.5000	-
Hinstling- Brounstein	1 − 2x/3 − (1 − x)^2/3^	0–12.5	600	0.7716	0.6484	650	0.8538	0.7563
		12.5–90	600	0.4211	-	650	0.5693	-
Shrinking sphere	1 − (1 − x)^1/3^	0–12.5	600	0.9562	0.8599	650	0.9802	0.9569
		12.5–90	600	0.6074	-	650	0.7959	-
Erofeev-Avrami *n* = 2	[−ln(1 − x)]^1/2^	0–12.5	600	0.9738	0.9906	650	0.9644	0.9741
		12.5–90	600	0.8030	-	650	0.9691	-
Erofeev-Avrami *n* = 3	[−ln(1 − x)]^1/3^	0–12.5	600	0.8333	0.9139	650	0.8331	0.8361
		12.5–90	600	0.9837	-	650	0.9653	-
Erofeev-Avrami *n* = 4	[−ln(1 − x)]^1/4^	0–12.5	600	0.6968	0.5004	650	0.7122	0.7138
		12.5–90	600	0.9571	-	650	0.8578	-
Chemical reaction of first order	−ln(1 − x)	0–12.5	600	0.9077	0.7469	650	0.9578	0.9125
		12.5–90	600	0.4351	-	650	0.6597	-
Chemical reaction of second order	(1 − x)^−1^ − 1	0–12.5	600	0.7278	0.4778	650	0.8639	0.7578
		12.5–90	600	0.2317	-	650	0.4316	-

**Table 8:** Parameters ^1^ of the illustrated in Figure 14 Mössbauer spectra of ZPR roasted with the addition of 48% Fe_2_(SO_4_)_3_∙9H_2_O during 180 min at 600 °C, as well as of the same sample after water leaching.

Temperature of Spectra Collecting, K			296					78				
Sample	Subspectrum, #	Phase	δ	Δ = 2ε	Γ_exp_	H_eff_	S	δ	Δ = 2ε	Γ_exp_	H_eff_	S
			mm/s	mm/s	mm/s	kOe	%	mm/s	mm/s	mm/s	kOe	%
Roasted	1	α-Fe_2_O_3_	0.37	−0.18	0.32	513.3	21.8	0.48	0.33	0.33	539.6	18
	2	γ-Fe_2_O_3_	0.31	−0.01	0.55	488.2	23	0.47	0.05	0.41	526.5	17
	3		0.32	−0.08	1.17	441	17	0.41	−0.03	0.67	508.3	29
	4	FeSO_4_∙H_2_O	1.26	2.72	0.47	-	3.6	1.37	3.12	0.71	-	5.3
	5	Fe_2_O(SO_4_)_2_∙xH_2_O	0.40	0.60	0.63	-	21	0.50	0.61	0.57	-	21
	6	ZnFe_2_O_4_	0.35	0.41	0.29	-	14	0.46	0.37	0.30	-	10
Leached	1	α-Fe_2_O_3_	0.37	−0.19	0.32	513.9	31.4	0.48	0.38	0.30	539.7	27
	2	γ-Fe_2_O_3_	0.33	−0.03	0.72	486.8	26	0.47	0.02	0.53	527.2	30
	3		0.34	−0.03	1.77	438	19	0.41	0.01	0.74	505	24
	6	ZnFe_2_O_4_	0.35	0.44	0.35	-	11.3	0.44	0.41	0.32	-	9
	7	Fe^+3^_Oh_	0.33	-	1.77	-	11.9	0.44	-	2.0	-	11

^1^ δ—isomer shift; Δ = 2ε—quadrupole splitting; Γ_exp_—line width; H_eff_—hyperfine magnetic field; S—relative area of a subspectrum #.

**Figure A1:**
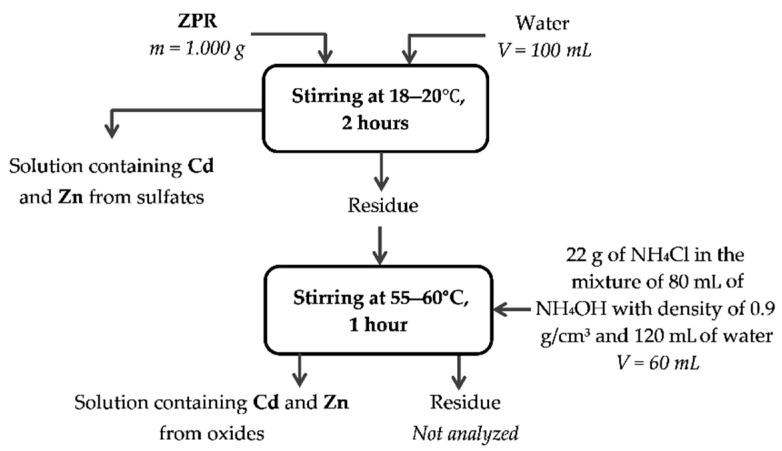
Method of chemical phase analysis of cadmium and zinc compounds.

**Figure A2:**
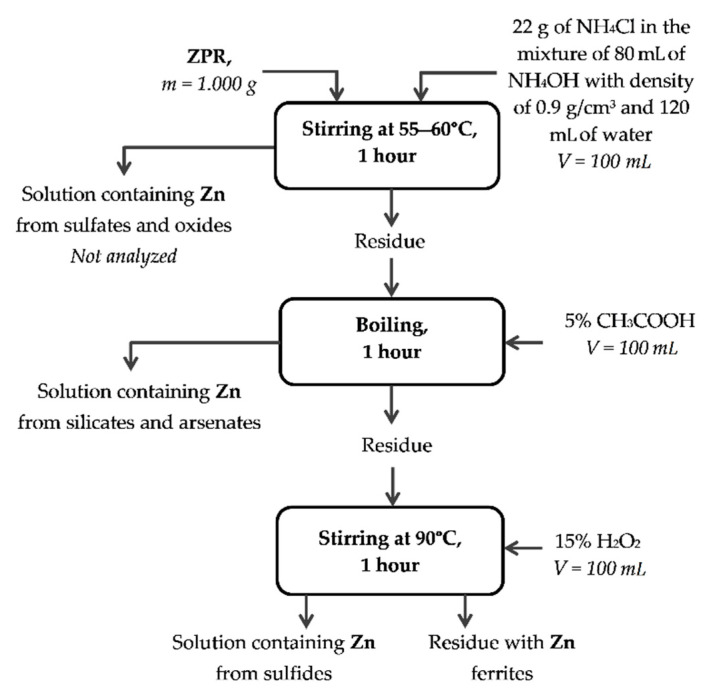
Method of chemical phase analysis of zinc compounds.

**Figure A3:**
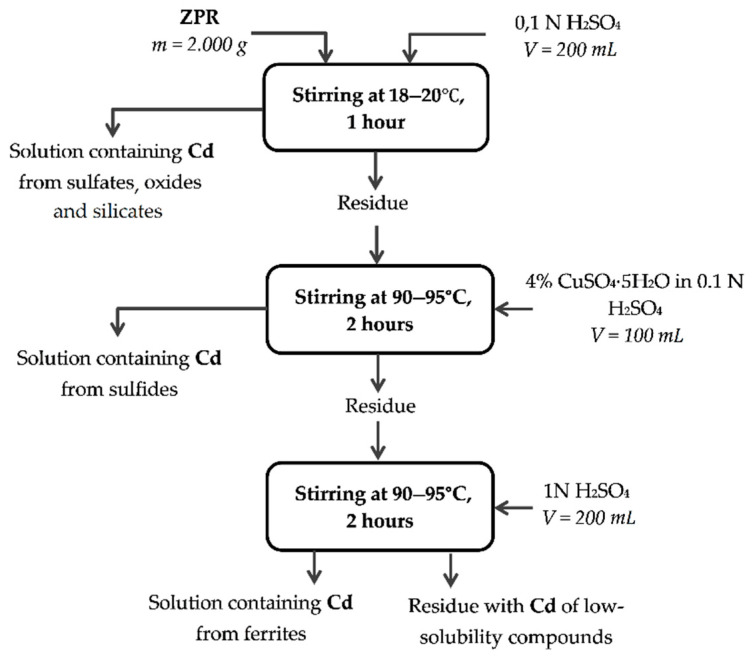
Method of chemical phase analysis of cadmium compounds.

**Figure A5:**
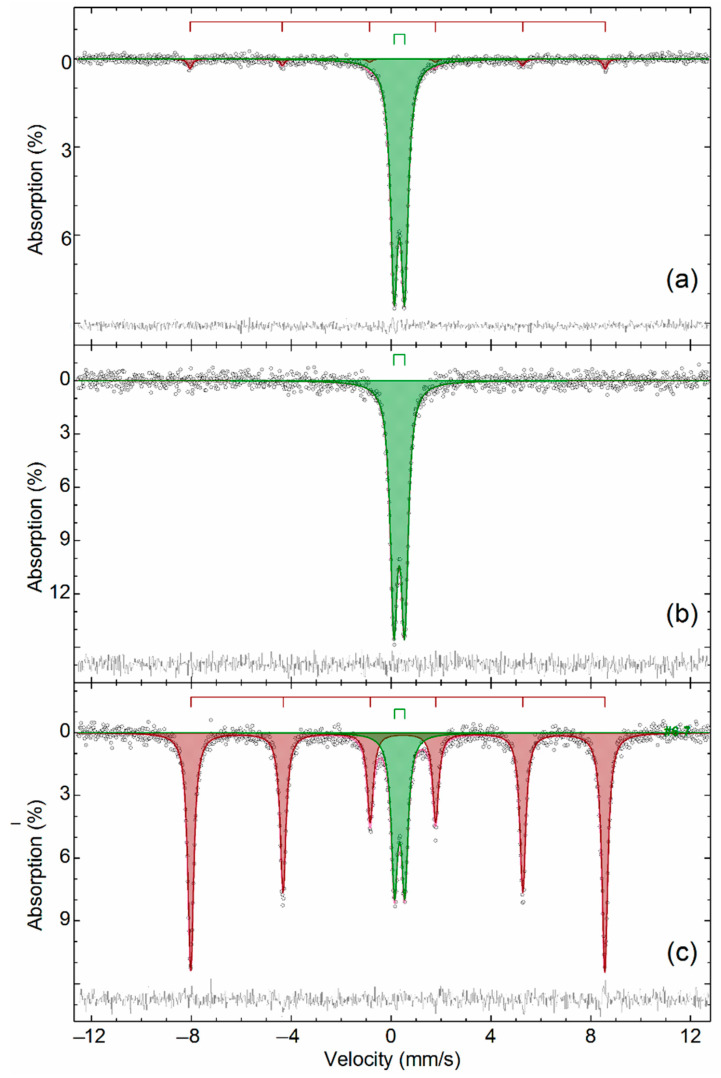
The Mössbauer spectra obtained at 296 K of ZPR roasted: (**a**) without additions at 600 °C; (**b**) with addition of 48% Fe_2_(SO_4_)_3_ at 600 °C, then additionally calcined at 900 °C; (**c**) with addition of 48% Fe_2_(SO_4_)_3_ at 600 °C, water-leached, then additionally calcined at 900 °C.

## Data Availability

The data presented in this study are openly available in Mendeley Data at DOI: 10.17632/67g57bj4kv.1, reference number [65], as well as at DOI: 10.17632/tvc8rcrdvz.1, reference number [103].

## Appendix A

Figure A1, Figure A2, Figure A3 and Figure A4 illustrate schematic diagrams of methods of chemical phase analysis developed for this paper based on the information from [49].

## Appendix B

Figure A5 and Table A1 show the supplementary Mössbauer spectra and their parameters, respectively, which were obtained to clarify and confirm phase identification of the main spectra. All the raw data for the main and supplementary spectra can be found in dataset [103].

**Table A1:** Parameters ^1^ of the Mössbauer spectra collected at 296 K of ZPR samples treated at different conditions; roasting and calcination durations were 180 min.

Sample	Phase	δ	Δ = 2ε	Γ_exp_	H_eff_	S
		mm/s	mm/s	mm/s	kOe	%
ZPR roasted without additions at 600 °C	α-Fe_2_O_3_	0.36	−0.20	0.22	515.8	5.5
	ZnFe_2_O_4_	0.33	0.42	0.36	-	94.5
ZPR roasted with addition of 48% Fe_2_(SO_4_)_3_ at 600 °C, then additionally calcined at 900 °C	ZnFe_2_O_4_	0.33	0.43	0.36	-	100
ZPR roasted with addition of 48% Fe_2_(SO_4_)_3_ at 600 °C, water-leached, then additionally calcined at 900 °C	α-Fe_2_O_3_	0.37	−0.21	0.29	514.5	75.2
	ZnFe_2_O_4_	0.34	0.41	0.32	-	24.8

^1^ δ—isomer shift; Δ = 2ε—quadrupole splitting; Γ_exp_—line width; H_eff_—hyperfine magnetic field; S—relative area of a subspectrum.

## References

[1] Wills B.A., Finch J.A. (2016). Appendix I—Metallic Ore Minerals. Wills’ Mineral Processing Technology. An Introduction to the Practical Aspects of Ore Treatment and Mineral Recovery.

[2] Mitra S. (2018). Depletion, technology, and productivity growth in the metallic minerals industry. Miner. Econ..

[3] Agrawal A., Sahu K., Pandey B.D. (2004). Solid waste management in non-ferrous industries in India. Resour. Conserv. Recycl..

[4] Vodyanitskii Y.N., Minkina T.M., Kubrin S., Pankratov D.A., Fedorenko A.G. (2019). Common and rare iron, sulfur, and zinc minerals in technogenically contaminated hydromorphic soil from Southern Russia. Environ. Geochem. Health.

[5] Sahu S.K., Razi M.K., Beuscher M., Chagnes A. (2020). Recovery of Metal Values from Ni-Cd Cake Waste Residue of an Iranian Zinc Plant by Hydrometallurgical Route. Metals.

[6] Sinclair R.J. (2005). The Extractive Metallurgy of Zinc.

[7] Monhemius A. (2017). The iron elephant: A brief history of hydrometallurgists’ struggles with element no.26. CIM J..

[8] Vignes A. (2011). Extractive Metallurgy 2: Metallurgical Reaction Processes.

[9] Kozlov P.A. (2003). The Waelz process.

[10] Stoychev S., Minchev E., Kyurkchiev A., Radonov G., Siegmund A., Alam S., Grogan J., Kerney U., Shibata E. (2020). Technologies for Treatment of Zinc-Containing Waste from Metallurgy in KCM AD. PbZn 2020: 9th International Symposium on Lead and Zinc Processing.

[11] Rüşen A., Topçu M.A. (2017). Investigation of zinc extraction from different leach residues by acid leaching. Int. J. Environ. Sci. Technol..

[12] Xing P., Ma B.-Z., Zeng P., Wang C.-Y., Wang L., Zhang Y.-L., Chen Y.-Q., Wang S., Wang Q.-Y. (2017). Deep cleaning of a metallurgical zinc leaching residue and recovery of valuable metals. Int. J. Miner. Met. Mater..

[13] Xie T.F., Sun C.Y., Li G.J., Luo Y.G., Zheng X.M., Ma A.Y., Li J., Zhang M.M., Li B.W., Monteiro S.M., Ikhmayies S., Kalay Y.E., Hwang J.-Y., Escobedo-Diaz J.P., Carpenter J.S., Brown A.D. (2021). Zinc Extraction from Industrial Waste Residue by Conventional Acid Leaching. Characterization of Minerals, Metals, and Materials.

[14] Vahidi E., Rashchi F., Moradkhani D. (2008). Recovery of zinc from an industrial zinc leach residue by solvent extraction using D2EHPA. Miner. Eng..

[15] Ashtari P., Pourghahramani P. (2015). Selective mechanochemical alkaline leaching of zinc from zinc plant residue. Hydrometallurgy.

[16] Huang Y., Geng Y., Han G., Cao Y., Peng W., Zhu X., Zhang T.-A., Dou Z. (2020). A perspective of stepwise utilization of hazardous zinc plant purification residue based on selective alkaline leaching of zinc. J. Hazard. Mater..

[17] Liu W., Sun S., Zhang L., Jahanshahi S., Yang J. (2012). Experimental and simulative study on phase transformation in Bayer red mud soda-lime roasting system and recovery of Al, Na and Fe. Miner. Eng..

[18] Guo Z.-H., Pan F.-K., Xiao X.-Y., Zhang L., Jiang K.-Q. (2010). Optimization of brine leaching of metals from hydrometallurgical residue. Trans. Nonferrous Met. Soc. China.

[19] Fan Y., Liu Y., Niu L., Zhang W., Zhang T.-A. (2021). High purity metal lead recovery from zinc direct leaching residue via chloride leaching and direct electrolysis. Sep. Purif. Technol..

[20] Palden T., Regadio M., Onghena B., Binnemans K. (2019). Selective Metal Recovery from Jarosite Residue by Leaching with Acid-Equilibrated Ionic Liquids and Precipitation-Stripping. ACS Sustain. Chem. Eng..

[21] Rodriguez N.R., Machiels L., Onghena B., Spooren J., Binnemans K. (2020). Selective recovery of zinc from goethite residue in the zinc industry using deep-eutectic solvents. RSC Adv..

[22] Yan H., Chai L.-Y., Peng B., Li M., Liu W., Peng N., Hou D.-K. (2013). Reduction Roasting of High Iron-Bearing Zinc Calcine under a CO-CO2 Gas: An Investigation of the Chemical and Mineralogical Transformations. JOM.

[23] Han J., Liu W., Qin W., Peng B., Yang K., Zheng Y. (2014). Recovery of zinc and iron from high iron-bearing zinc calcine by selective reduction roasting. J. Ind. Eng. Chem..

[24] Wang C., Guo Y.-F., Wang S., Chen F., Tan Y.-J., Zheng F.-Q., Yang L.-Z. (2020). Characteristics of the reduction behavior of zinc ferrite and ammonia leaching after roasting. Int. J. Miner. Met. Mater..

[25] Kashyap V., Taylor P. (2020). Selective Extraction of Zinc from Zinc Ferrite. Min. Met. Explor..

[26] Zheng Y.-X., Lv J.-F., Liu W., Qin W.-Q., Wen S.-M. (2016). An innovative technology for recovery of zinc, lead and silver from zinc leaching residue. Physicochem. Probl. Miner. Process..

[27] Min X.-B., Jiang G.-H., Wang Y.-Y., Zhou B.-S., Xue K., Ke Y., Xu Q.-J., Wang J.-W., Ren H.-C. (2020). Sulfidation roasting of zinc leaching residue with pyrite for recovery of zinc and iron. J. Central South Univ..

[28] Holloway P.C., Etsell T.H., Murland A.L. (2007). Roasting of La Oroya Zinc Ferrite with Na_2_CO_3_. Met. Mater. Trans. A.

[29] Holloway P.C., Etsell T.H., Murland A.L. (2007). Use of Secondary Additives to Control the Dissolution of Iron during Na2CO3 Roasting of La Oroya Zinc Ferrite. Met. Mater. Trans. A.

[30] Holloway P.C., Etsell T.H. (2012). Recovery of zinc, gallium and indium from La Oroya zinc ferrite using Na_2_CO_3_ roasting. Miner. Process. Extr. Metall..

[31] Youcai Z., Stanforth R. (2000). Extraction of zinc from zinc ferrites by fusion with caustic soda. Miner. Eng..

[32] Wang H.B., Zheng C.Z., Qin S.C., Siegmund A., Alam S., Grogan J., Kerney U., Shibata E. (2020). Study of a Novel Chloride Volatilization Process for the Treatment of Jarosite Residue. Proceedings of the PbZn 2020: 9th International Symposium on Lead and Zinc Processing.

[33] Zhang Y., Yu X., Li X. (2011). Zinc recovery from franklinite by sulphation roasting. Hydrometallurgy.

[34] Güler E., Seyrankaya A., Cöcen İ. (1999). Effect of Sulfation Roasting on Metal Extraction from Çinkur Zinc Leach Residue. J. Ore Dress..

[35] Turan M.D., Altundoğan H.S., Tümen F. (2004). Recovery of zinc and lead from zinc plant residue. Hydrometallurgy.

[36] Wang R.-X., Yang Y.-D., Liu C.-X., Zhou J., Fang Z., Yan K., Tian L., Xu Z.-F. (2020). Recovery of lead and silver from zinc acid-leaching residue via a sulfation roasting and oxygen-rich chlorination leaching method. J. Central South Univ..

[37] Li Y., Liu H., Peng B., Min X., Hu M., Peng N., Yuang Y., Lei J. (2015). Study on separating of zinc and iron from zinc leaching residues by roasting with ammonium sulphate. Hydrometallurgy.

[38] Fekete F., Lázár K., Keszler A.M., Jánosity A., Zhibin L., Szilágyi I.M., Kótai L. (2018). Recycling the industrial waste ZnFe2O4 from hot-dip galvanization sludge. J. Therm. Anal. Calorim..

[39] De Oliveira C.C.S., Pereira D.D., Mendes F.R.P., Araujo M.F.L. (2020). A New Route for Treating Neutral Leaching Residue. Proceedings of the PbZn 2020: 9th International Symposium on Lead and Zinc Processing.

[40] Li Y.-C., Zhuo S.-N., Peng B., Min X.-B., Liu H., Ke Y. (2020). Comprehensive recycling of zinc and iron from smelting waste containing zinc ferrite by oriented transformation with SO_2_. J. Clean. Prod..

[41] De Oliveira C.C.S., Pereira D.D. (2020). Simulation of an Alternative Direct Leaching Process for High Iron Content Zinc Concentrates. Proceedings of the PbZn 2020: 9th International Symposium on Lead and Zinc Processing.

[42] Hu M., Peng B., Chai L.-Y., Li Y.-C., Peng N., Yuan Y.-Z., Chen N. (2015). High-Zinc Recovery from Residues by Sulfate Roasting and Water Leaching. JOM.

[43] Jiang G.-M., Peng B., Liang Y.-J., Chai L.-Y., Wang Q.-W., Li Q.-Z., Hu M. (2017). Recovery of valuable metals from zinc leaching residue by sulfate roasting and water leaching. Trans. Nonferrous Met. Soc. China.

[44] Altundoǧan H., Tümen F. (1997). Metal recovery from copper converter slag by roasting with ferric sulphate. Hydrometallurgy.

[45] Nadirov R.K. (2018). Recovery of Valuable Metals from Copper Smelter Slag by Sulfation Roasting. Trans. Indian Inst. Met..

[46] Grudinsky I.P., Podjelnikova E.S., Dyubanov V.G. (2020). Research on the Process of Sulphatizing Roasting of Copper Slag Flotation Tailings Using Iron Sulphates. IOP Conference Series: Earth and Environmental Science.

[47] Grudinsky I.P., Zhiltsova E.E., Grigorieva D.D., Dyubanov V.G. (2021). Experimental Study of the Sulphatizing Roasting of Flotation Tailings from Copper Slag Processing Using Iron Sulfates. IOP Conference Series: Earth and Environmental Science.

[48] Pickles C.A., Marzoughi O. (2018). Thermodynamic Investigation of the Sulphation Roasting of Electric Arc Furnace Dust. Minerals.

[49] Filippova N.A. (1975). The Phase Analysis of Ores and Their Processing Products.

[50] Talanov V., Shabelskaya N., Golovina A. (2010). Method of copper ferrite preparation. Russia Patent.

[51] (2021). Match!. Software for Phase Analysis Using Powder Diffraction.

[52] HSC Chemistry (2019). Software for Chemical Reaction and Equilibrium Calculation.

[53] Kovalev D.Y., Ponomarev V.I. (2019). Time-Resolved X-Ray Diffraction in SHS Research and Related Areas: An Overview. Int. J. Self-Propag. High-Temp. Synth..

[54] Pankratov D.A. (2013). Mössbauer study of oxo derivatives of iron in the Fe_2_O_3_-Na_2_O_2_ system. Inorg. Mater..

[55] König U., Bertaut E., Gros Y., Mitrikov M., Chol G. (1970). Models of the magnetic structure of zinc ferrite. Solid State Commun..

[56] Li F., Wang L., Wang J., Zhou Q., Zhou X., Kunkel H., Williams G. (2004). Site preference of Fe in nanoparticles of ZnFe2O4. J. Magn. Magn. Mater..

[57] Chinnasamy C.N., Narayanasamy A., Ponpandian N., Chattopadhyay K.K., Guérault H., Greneche J.-M. (2000). Magnetic properties of nanostructured ferrimagnetic zinc ferrite. J. Phys. Condens. Matter.

[58] Sato T., Haneda K., Seki M., Iijima T. (1990). Morphology and magnetic properties of ultrafine ZnFe_2_O_4_ particles. Appl. Phys. A.

[59] Evans B.J., Hafner S.S., Weber H.P. (1971). Electric Field Gradients at ^57^Fe in ZnFe_2_O_4_ and CdFe_2_O_4_. J. Chem. Phys..

[60] Varshney D., Verma K., Kumar A. (2011). Structural and vibrational properties of ZnxMn1−xFe_2_O_4_ (x = 0.0, 0.25, 0.50, 0.75, 1.0) mixed ferrites. Mater. Chem. Phys..

[61] Waerenborgh J.C., Figueiredo M.O., Cabral J., Pereira L. (1994). Temperature and Composition Dependence of the Cation Distribution in Synthetic ZnFeyAl_2_-yO_4_ (0 ≤ y ≤ 1) Spinels. J. Solid State Chem..

[62] Toledo J., Valenzuela M., Bosch P., Armendáriz H., Montoya A., Nava N., Vázquez A. (2000). Effect of AI3+ introduction into hydrothermally prepared ZnFe_2_O_4_. Appl. Catal. A Gen..

[63] Valeev D., Zinoveev D., Kondratiev A., Lubyanoi D., Pankratov D. (2019). Reductive Smelting of Neutralized Red Mud for Iron Recovery and Produced Pig Iron for Heat-Resistant Castings. Metals.

[64] Nininger R.C., Schroeer D. (1978). Mössbauer studies of the morin transition in bulk and microcrystalline α-Fe2O3. J. Phys. Chem. Solids.

[65] Grudinsky P. (2021). TG-DSC raw data and plots for the mixtures containing ferrites (MFe2O4, where M = Zn, Cu or Zn0.5Cu0.5) and iron sulfates (Fe_2_(SO_4_)_3_∙9H_2_O or FeSO_4_∙7H_2_O).

[66] Pelovski Y., Petkova V., Nikolov S. (1996). Study of the mechanism of the thermochemical decomposition of ferrous sulphate monohydrate. Thermochim. Acta.

[67] Swami M.S.R., Prasad T.P. (1980). Thermal analysis of iron(II) sulphate heptahydrate in air. J. Therm. Anal. Calorim..

[68] Saini A., Kótai L., Sajo I.E., Szilagyi I.M., Lázár K., May Z., Fazekas P., Gács I., Sharma V., Banerji K.K. (2012). Solid phase sulphatizing of zinc ferrite spinel with iron sulphates as an environmental friendly way for recovering zinc. Eur. Chem. Bull..

[69] Dutrizac J.E., Macdonald R.J.C., Lamarche R.E. (1975). Solubility of silver sulfate in acidified ferric sulfate solutions. J. Chem. Eng. Data.

[70] Lide D.R. (2005). Physical constants of inorganic compounds. CRC Handbook of Chemistry and Physics.

[71] Liu Q., Tan J., Liu C.Q., Yin Z.L., Chen Q.Y., Liao Z., Xie F.C., Zhang P.M. (2014). Thermodynamic study of metal sulfate de-composition process in bath smelting. Chin. J. Nonferrous Met..

[72] Fedorov P.P., Proidakova V.Y., Kuznetsov S., Voronov V. (2017). Phase equilibria in systems of gallium sulfate with lithium or sodium sulfate. Russ. J. Inorg. Chem..

[73] Stern K.H. (2001). Thermal Decomposition of Inorganic Salts with Oxyanions.

[74] Guseva A.F., Neyman A.Y., Animitsa I.E. (2005). Solid Phase Reactions during the Production and Exploitation of Inorganic Materials.

[75] Rostovshchikova T., Smirnov V.V., Tsodikov M.V., Bukhtenko O.V., Maksimov Y.V., Kiseleva O.I., Pankratov D. (2005). Catalytic conversions of chloroolefins over iron oxide nanoparticles 1. Isomerization of dichlorobutenes in the presence of iron oxide nanopaticles immobilized on silicas with different structures. Russ. Chem. Bull..

[76] Rostovshchikova T.N., Korobov M.S., Pankratov D.A., Yurkov G., Gubin S.P. (2005). Catalytic conversions of chloroolefins over iron oxide nanoparticles 2. Isomerization of dichlorobutenes over iron oxide nanoparticles stabilized on the surface of ultradispersed poly(tetrafluoroethylene). Russ. Chem. Bull..

[77] Choi H., Seo J.Y., Kim J., Kim C.S., Uhm Y.R., Sun G.M. (2020). Standard Iron Oxides and M$ddot{o}$ssbauer Spectroscopy. New Phys. Sae Mulli.

[78] Oh S.J., Cook D., Townsend H. (1998). Characterization of Iron Oxides Commonly Formed as Corrosion Products on Steel. Hyperfine Interact..

[79] Pankratov D.A., Anuchina M.M., Spiridonov F.M., Krivtsov G.G. (2020). Fe3—δO4 Nanoparticles Synthesized in the Presence of Natural Polyelectrolytes. Crystallogr. Rep..

[80] Goya G., Rechenberg H. (1998). Superparamagnetic transition and local disorder in CuFe2O4 nanoparticles. Nanostruct. Mater..

[81] Amir M., Gungunes H., Slimani Y., Tashkandi N., El Sayed H.S., Aldakheel F., Sertkol M., Sözeri H., Manikandan A., Ercan I. (2018). Mössbauer Studies and Magnetic Properties of Cubic CuFe_2_O_4_ Nanoparticles. J. Supercond. Nov. Magn..

[82] Chatterjee B.K., Bhattacharjee K., Dey A., Ghosh C.K., Chattopadhyay K.K. (2014). Influence of spherical assembly of copper ferrite nanoparticles on magnetic properties: Orientation of magnetic easy axis. Dalton Trans..

[83] Cross W.B., Affleck L., Kuznetsov M.V., Parkin I., Pankhurst Q.A. (1999). Self-propagating high-temperature synthesis of ferrites MFe_2_O_4_ (M = Mg, Ba, Co, Ni, Cu, Zn); reactions in an external magnetic field. J. Mater. Chem..

[84] Patil V., Kulkarni R. (1979). Magnetic properties of Cu-Zn ferrite investigated by Mössbauer spectroscopy. Solid State Commun..

[85] Siddique M., Khan R.T.A., Shafi M. (2008). Fluctuation in occupancy of Cu^2+^ ions in Zn- and Cd-substituted Cu-ferrites. J. Radioanal. Nucl. Chem..

[86] Ata-Allah S., Hashhash A. (2006). Jahn–Teller effect and superparamagnetism in zn substituted copper-gallate ferrite. J. Magn. Magn. Mater..

[87] Modi K.B., Shah S., Kathad C.R., Dulera S.V., Jethvani B.B., Popat M.V., Lakhani V.K., Chandra U. (2014). Study on Mössbauer Signature, Hyperfine Interaction Parameters and Removal of Delafossite Phase of Al3+-Modified Copper Ferrite. J. Supercond. Nov. Magn..

[88] Van Alboom A., De Resende V.G., De Grave E., Gómez J.A.M. (2009). Hyperfine interactions in szomolnokite (FeSO_4_·H_2_O). J. Mol. Struct..

[89] Dékány I., Turi L., Homonnay Z., Vértes A., Burger K. (1996). Preparation of nanosize FeS particles on SiO_2_ and clay mineral supports: SAXS and Mössbauer spectroscopic measurements. Colloids Surf. A Physicochem. Eng. Asp..

[90] Zboril R., Mashlan M., Petridis D., Krausova D., Pikal P. (2002). The Role of Intermediates in the Process of Red Ferric Pigment Manufacture from FeSO_4_⋅7H_2_O. Hyperfine Interact..

[91] Ristic M., Music S., Orehovec Z. (2005). Thermal decomposition of synthetic ammonium jarosite. J. Mol. Struct..

[92] Haven Y., Noftle R.E. (1977). The Mössbauer isomer shift in iron (III) sulfate. J. Chem. Phys..

[93] Majzlan J., Alpers C.N., Koch C.B., McCleskey R.B., Myneni S.C., Neil J.M. (2011). Vibrational, X-ray absorption, and Mössbauer spectra of sulfate minerals from the weathered massive sulfide deposit at Iron Mountain, California. Chem. Geol..

[94] Portov A.B., Ozerov S.S., Tsymbulov L.B., Mashyanov A.K. (2016). Usage of sulfuric acid and iron sulfate solutions, copper, and nickel as binders in briquetting of an ore copper-nickel concentrate. Tsvetnye Met..

[95] Olson E.S. (1998). Binder Modification and Development for Briquetting Steel Mill Residues.

[96] Kawatra S.K., Eisele T.C., Lewandowski K.A., Gurtler J.A. (2006). Novel Binders and Methods for Agglomeration of Ore.

[97] Shi Y., Fan M. (2007). Reaction Kinetics for the Catalytic Oxidation of Sulfur Dioxide with Microscale and Nanoscale Iron Oxides. Ind. Eng. Chem. Res..

[98] Wu Z., Li X., Chen J., Hu H., Chen D., Yao H. The effect of minerals in coal ash on the formation of SO_3_ in flue gas. Proceedings of the International Conference on Power Engineering (ICOPE).

[99] Fu H., Wang X., Wu H., Yin Y., Chen J. (2007). Heterogeneous Uptake and Oxidation of SO_2_ on Iron Oxides. J. Phys. Chem. C.

[100] Kolmachikhina E.B., Sviridov A., Naumov K.D. (2020). Investigation into the Influence of Sodium Lignosulfonate, Anionic Surfactants, and Their Mixtures on the Copper Cementation Rate by Zinc. Russ. J. Non-Ferrous Met..

[101] Chen C.-S., Shih Y.-J., Huang Y.-H. (2016). Recovery of lead from smelting fly ash of waste lead-acid battery by leaching and electrowinning. Waste Manag..

[102] Badanoiu G., Buzatu T., Ghica V.G., Buzatu M., Iacob G., Petrescu I.M. (2014). Study of PbSO_4_ solubilisation in NaOH solution, for the treatment of oxide-sulphate pastes obtained from dismembered lead-acid batteries. UPB Sci. Bull. Ser. B Chem. Mater. Sci..

[103] Pankratov D.A., Grudinsky P.I. (2021). Mossbauer spectra raw data for article “Comprehensive Study on the Mechanism of Sulfating Roasting of Zinc Leaching Residue with Iron Sulfates; Mendeley data” Version 1. Materials.

